# Impaired Glymphatic Transport Kinetics Following Induced Acute Ischemic Brain Edema in a Mouse pMCAO Model

**DOI:** 10.3389/fneur.2022.860255

**Published:** 2022-03-17

**Authors:** Jianying Zhang, Hongchen Zhao, Yang Xue, Yiqi Liu, Guohang Fan, He Wang, Qiang Dong, Wenjie Cao

**Affiliations:** ^1^Department of Neurology, Huashan Hospital, Fudan University, Shanghai, China; ^2^The Key Laboratory of Computational Neuroscience and Brain-Inspired Intelligence, Institute of Science and Technology for Brain-Inspired Intelligence, Fudan University, Shanghai, China; ^3^State Key Laboratory of Medical Neurobiology, Fudan University, Shanghai, China; ^4^National Clinical Research Center for Aging and Medicine, Huashan Hospital, Fudan University, Shanghai, China

**Keywords:** magnetic resonance imaging, glymphatic, aquaporin-4, cerebral ischemic edema, polarization

## Abstract

**Background:**

Cerebral edema forms immediately after blood flow interruption in ischemic stroke, which largely increased the death and disability. The glymphatic (glial-lymphatic) pathway is a major regulator of the brain liquid dynamics and homeostasis. This study aimed to investigate the transport kinetics of the glymphatic system after the appearance of ischemic edema.

**Methods:**

In this study, a coated filament was attached to the left middle cerebral artery (MCA) of mice to establish a mouse model of permanent middle cerebral artery occlusion with an intact blood-brain barrier (BBB). The glymphatic function was then quantified using contrast-enhanced MRI (11.7T) by employing an injection of gadobenate dimeglumine (BOPTA-Gd) into the cisterna magna of mice. We then evaluated the expression and polarization of aquaporin-4 (AQP4) as a proxy for the physiological state of the glymphatic system.

**Results:**

Our results revealed a positive correlation between the signal intensity in T1-weighted images and the corresponding apparent diffusion coefficient (ADC) values in the cortex, striatum, and periventricular zone, suggesting that impaired glymphatic transport kinetics in these regions is correlated to the cytotoxic edema induced by the occlusion of MCA. Furthermore, the increased depolarization of AQP4 in the parenchyma perivascular space (PVS) was consistent with glymphatic failure following the induced early cerebral ischemic edema.

**Conclusions:**

Glymphatic transport kinetics were suppressed between the onset of cytotoxic edema and the disruption of the BBB, which correlated with the diminishing ADC values that vary based on edema progression, and is associated with depolarization of AQP4 in the parenchyma PVSs.

## Introduction

The severity of cerebral edema following ischemic stroke is predictive of a patient's functional outcome (1). However, current treatment options are often ineffective. Ischemic cerebral edema occurs soon after the cerebral blood flow (CBF) interruption, which can lead to severe stroke-related damage (2). This phenomenon is further exacerbated if the edema is aggravated and can lead to long-term or permanent effects even after the vessels are successfully re-canalized. In patients with early postischemia neurological deterioration, the effect of cerebral edema on the clinical outcome of reperfusion therapy is generally more pronounced (3). Cerebral edema after stroke has mainly been thought to be caused by the destruction of the blood-brain barrier (BBB) (4), which can allow blood components to penetrate this boundary, enter the parenchyma, and damage the tissue. Because of this, previous studies investigating the mechanism of cerebral edema have largely focused on the destruction of the BBB (5). Nevertheless, recent reports showed that cerebral edema develops several hours before significant dysfunction of the BBB (6), which posed a new question regarding other influential factors that arise cerebral ischemia edema. In fact, cytotoxic edema begins within minutes of ischemic destruction and is triggered by spreading depolarizations (SDs). These traveling neurophysiological events form a dysregulated ion homeostasis that often results in cell swelling (7), and are often accompanied by ionic edema and the net entry of water and ions into the brain (8), defined as interstitial edema. Based on this series of evidence, there exists a key time window between the onset of cytotoxic edema and the disruption of the BBB in which the transformation of cytotoxic edema to vasogenic edema occurs.

A previous study verified that an increased glymphatic influx of cerebrospinal fluid (CSF) into the parenchyma perivascular spaces (PVSs) (9) of ischemic brain tissue might be the actual cause of superacute ischemic brain edema (10). CSF influx was observed at (11.4 ± 1.8 s) and (5.24 ± 0.48 min), and was driven by SDs in the cortex following cerebral ischemia and led to edema (10). We then asked in a follow-up study how the kinetics of glymphatic functions were affected after the influx of the CSF. This influx occurred through the glymphatic system which is a brain-wide mass clearance system (11) that functions through fluid transport and has been confirmed in both humans (12) and mice (9, 13). In particular, the PVSs by which the CSF and interstitial fluid (ISF) exchange occurs act as flow channels that ensheath the cerebral vasculature in the mammalian brain (9). Aquaporin-4 (AQP4) is an essential water channel protein that facilitates this process through a complex network of astrocytes (14) that are localized throughout brain tissues facing the PVS (15) and ventricle lining (16). The glymphatic system, therefore, supports convective bulk flow (9, 14) by astrocytic paracellular fluid transport and facilitates the drainage of brain ISF between para-arterial influx and paravenular efflux. We hypothesis this process may facilitate brain swelling by glymphatic malfunctions.

In this study, we aimed to explore the alteration in glymphatic dynamics after the accumulation of fluid in the brain parenchyma and periventricular zone. We also assessed glymphatic system dysfunction and its correlation with parenchymal and periventricular edema based on the signal intensity (SI) in T1-weighted images (T1WIs) and the corresponding apparent diffusion coefficient (ADC) value and further explored the altered polarization patterns of AQP4 in various affected edema regions in permanent middle cerebral artery occlusion (pMCAO) mice. We quantified the kinetics of glymphatic drainage using 11.7T contrast-enhanced magnetic resonance imaging (CEMRI) with intracisternal injections of BOPTA-Gd (17) after the onset of ischemic edema in a mouse model of pMCAO without BBB disruption (18). The glymphatic functions of sham-operated and pMCAO mice were characterized along both spatial and temporal features to identify glymphatic malfunction in the edema mouse brain induced by occlusion of the MCA.

## Materials and Methods

The data that support the findings of this study are available from the corresponding author upon reasonable request and are subject to institutional agreements and ethical approvals. All experiments were reported in compliance with the ARRIVE guidelines 2.0 (ARRIVE, Animal Research: Reporting *in Vivo* Experiments) (19).

### Animals

All animal procedures were approved by the Huashan Hospital Institutional Review Board. The reason for using only male mice is that estrogen has been shown to significantly affect ischemic stroke (20). Male C57/BL6 mice (bodyweight 25–30 g) aged 10–12 weeks old were used in this study. Animals were housed at constant temperature (25°C ± 2°C) with a 12/12 h light/dark cycle and were provided with *ad libitum* access to food and water. Animals were randomly assigned to two groups using excel-generated random numbers: the sham-operated group or the pMCAO group. The experimenters were blinded to pretreatments and data analysis. This study was carried out per the recommendations of the National Science Council of the Republic of China. All efforts were made to minimize animal suffering and the number of experimental animals used.

### pMCAO Model

Previous studies investigating the mechanism of cerebral ischemic edema have mainly focused on the destruction of BBB (5). To do this, most experiments used an animal model of cerebral ischemia/reperfusion. By contrast, the BBB remains intact during permanent MCA occlusion (18), as indicated by continuous isolectin-B4 (IB4) staining (21), which allows for the mechanistic study of ischemic brain edema with an intact BBB. To perform pMCAO, mice were initially anesthetized with 4% isoflurane in 1 l/min oxygen delivered through a nose cone. The isoflurane was then adjusted to between 1.5 and 3% in 1 l/min oxygen to maintain anesthesia and normal breathing throughout the procedure. Animal body temperature was maintained at 37°C with a heating pad throughout the procedure. A laser Doppler probe was fixed on the skull 5 mm lateral to and 2 mm posterior to Bregma. A coated filament (1622A4, Cinontech Co. Ltd., Beijing, China) was then placed at the left MCA under a stereomicroscope. Concurrent CBF recordings were recorded by laser Doppler to ensure that it was below 25% of baseline. Only the mice presenting with MCAO on a 3D (three-dimensional)-TOF (time-of-flight) MRI performed immediately after surgery were included in subsequent analyses.

### Intracisternal Perfusion of Gadolinium-Based Contrast Agents

About 4 μl of BOPTA-Gd was injected into the cisterna magna with a microsyringe pump (ALC-IP600(H), ALCOTT, Shanghai, China) and a pulled glass micropipette (diameter = ~80 μm) that was connected and sealed to a 50 μl syringe with hot melt glue (19). This allowed us to perform a single diminutive volume injection of contrast agents without significant injury to the dura mater and, thus, without CSF leakage. The micropipette was left in place for 1 additional minute to allow the wound to close. The intracisternal injection was conducted at a flow rate of 0.6 μl/min over 6 min. About 28 min after the start of blood occlusion, the function of the glymphatic system and cerebral edema were evaluated by MRI.

### MRI Experiments and Imaging Analysis

Following surgery and cisterna magna injection, animals were transferred to an MRI-compatible cradle. About 1.5% isoflurane in oxygen was continuously delivered to the animal through a nose cone. The core body temperature of the mice was maintained at 37°C, and respiratory rate was maintained between 70 and 100 breaths per minute. Each MRI was acquired on an 11.7T Pharmascan MRI system (Bruker, Germany) equipped with surface coils. A baseline scan was acquired before intrathecal infusion of the paramagnetic contrast agent. 3D T1WI was then performed with a fast low angle shot sequence with parameters as follows: magnetic field strength = 11.75123799 Tesla, repetition time/echo time (TR/TE) 50/3.5 ms, field-of-view (FOV) = 16 mm ×16 mm, matrix = 128 ×128, slice thickness (ST) = 0.3 mm, flip angle (FA) = 15°. 3D-TOF imaging was performed with the following parameters: TR/TE 12/2.0 ms, field-of-view (FOV) = 16 mm ×16 mm, matrix = 120 ×120, ST = 0.13 mm, FA = 20°. T2-weighted (T2W) imaging was performed with the following parameters: TR/TE 2500/30 ms, FOV = 16 mm ×16 mm, matrix = 256 ×256, ST = 0.5 mm, FA = 90°. Finally, diffusion-weighted imaging (DWI) was performed with parameters set as follows: TR/TE 3200/23 ms, FOV = 16 mm ×16 mm, matrix = 180 ×180, ST = 0.3 mm, FA = 90°. The time course of distribution of the contrast agent throughout the brain was assessed as a measure of glymphatic flow, as demonstrated in a previous study (9, 22). The time-resolved T1W SI in the striatum, cortex, and cerebral ventricles were used to obtain the average hydrodynamic response curves. These time series were also used to illustrate the tissue uptake of BOPTA-Gd tracers over time. The ADC values were calculated automatically by the 11.7T Pharmascan MRI system and were displayed as a parametric map that indicated the degree of water molecule diffusion through different tissues. Then, ADC measurements were acquired for a given region by drawing regions of interest (ROIs) on the ADC map (23).

### Triphenyl Tetrazolium Chloride (TTC) Staining

Animals were anesthetized and perfused with 20 ml of ice-cold saline. After immediate decapitation, the brains were extracted and sliced into six 1.5 mm coronal sections and immersed into 2% 2,3,5-triphenyl tetrazolium chloride (TTC) (Sigma-Aldrich) for 10 min at room temperature. Images were then acquired with a digital camera.

### Section Preparation

About 150 min after surgery, the deeply anesthetized pMCAO animals (*n* = 4) and sham-operated animals (*n* = 4) were sacrificed. All animals were then transcardially perfused with ice-cold saline, followed by 4% paraformaldehyde (PFA). Brains were removed, postfixed in PFA overnight at 4°C, dehydrated in the ascending alcohol series, rinsed by xylene, and embedded in paraffin blocks. Coronal sections (bregma +0.38 mm to +1.10 mm) were serially cut at 3 μm thickness using a paraffin slicing machine (RM2135, Leica Microsystems, Germany).

### Immunofluorescence

After deparaffinization and hydration, antigen recovery was performed with ethylene diamine tetraacetic acid antigen retrieval buffer (pH 8.0). Coronal sections were maintained at a subboiling temperature for 8 min to facilitate staining with an AQP4 antibody. Sections were then blocked in phosphate-buffered saline (PBS) with 3% bovine serum albumin (BSA) for 30 min, and stained sequentially with primary mouse antibody AQP4 (1:500, #GB12529, Servicebio) and Alexa Fluor 594 antimouse (1:300, #GB21301, Servicebio), together with the fluorescein isothiocyanate (FITC) conjugate Bandeiraea simplicifolia Isolectin B4 (BSI-B4, 1:50, #L-2895, Sigma). The brain sections were then covered with an antifade medium containing 4′,6-diamidino-2-phenylindole (DAPI, #D21490, Invitrogen). For this experimental step, IB4-FITC (2 mg dissolved in 2 ml saline, 0.05 mg per animal) was intravenously administered to the animals to identify the basement membrane. This was an important step as IB4 binds to a-D-galactose residues in the basement membrane secreted by endothelial cells (24). In each group, the circulation period of IB4-FITC was ensured for 180 min before sacrifice.

### Immunohistochemistry

After deparaffinization, hydration, and antigen retrieval, sections were washed and incubated with the 3% hydrogen peroxide (H_2_O_2_) at room temperature for 15 min to block endogenous peroxidase. Then tissues were washed and blocked with 3% BSA at room temperature for 30 min. Tissue sections were incubated with primary mouse antibody AQP4 (1:2, 000, #GB12529, Servicebio) at 4°C overnight. Following PBS wash, sections were incubated with secondary antibody goat antimouse IgG (1:200, #GB23303, Servicebio) labeled with horseradish peroxidase, incubate at room temperature for 50 min. Then visualized with diaminobenzidine reagent (#G1211, Servicebio).

### Imaging Analysis

Microphotographs for analyzing immunofluorescence were captured by laser scanning confocal microscopy (FV1200, Olympus Microsystems, Japan) to generate 3-channel fluorescence images of the cerebral cortex and striatum. Images were obtained at 100 × magnification and quantified using the ImageJ software (Bethesda, MD, USA). For immunofluorescent quantification of each antigen, at least three selected visual fields (bregma +0.38 to +1.10 mm) from each animal were imaged. Imaging parameters were constant throughout all imaging sessions. The background fluorescence for each channel was conformably subtracted and the perivascular expression of AQP4 was then measured after that in the soma (“depolarization”) was subtracted.

Microphotographs for analyzing immunohistochemistry were captured by conventional fluorescence microscopy (BX60, Olympus Microsystems, Japan). For the AQP4 examination, at least three high-power fields at 100 × magnification were randomly selected and imaged for each animal. The ROI on the AQP4 display included the ependymal wall and a small amount of surrounding brain tissue in the bilateral brain of pMCAO animals. The average optical density (AOD) of AQP4 in each field was quantitatively calculated using the ImageJ software. All imaging parameters were constant throughout all imaging sessions.

### Quantification of AQP4 Expression and Polarization

To evaluate global and perivascular AQP4 expression levels, the mean AQP4 immunofluorescence intensity was measured within each visual field and the perivascular space. Evaluation of the polarization of AQP4 to perivascular end-feet was performed by measuring the immunofluorescence intensity of AQP4 immunoreactivity across sections of blood vessels labeled by IB4-FITC in each brain region studied. AQP4 polarization was measured by comparing the intensity ratios of AQP4 at perivascular domains vs. those in the parenchymal domains, as evaluated previously (25). The threshold was determined based on immunostained control slices.

### Statistical Analysis

Statistical analyses were conducted using Prism (GraphPad Software, V8.3.0, La Jolla, USA). The data are represented as mean ± SD. Either a repeated measure two-way ANOVA or a regular two-way ANOVA was used for regional comparisons, followed by Tukey's multiple comparisons test. A one-way ANOVA was used for regional comparisons, and unpaired *t*-tests were used for single comparisons. Pearson correlation analysis was utilized to evaluate the link between edema and glymphatic failure. A *p*-value < 0.05 was considered statistically significant.

## Results

### Occlusion of Blood Flow in pMCAO Mice

**Figure 1:**
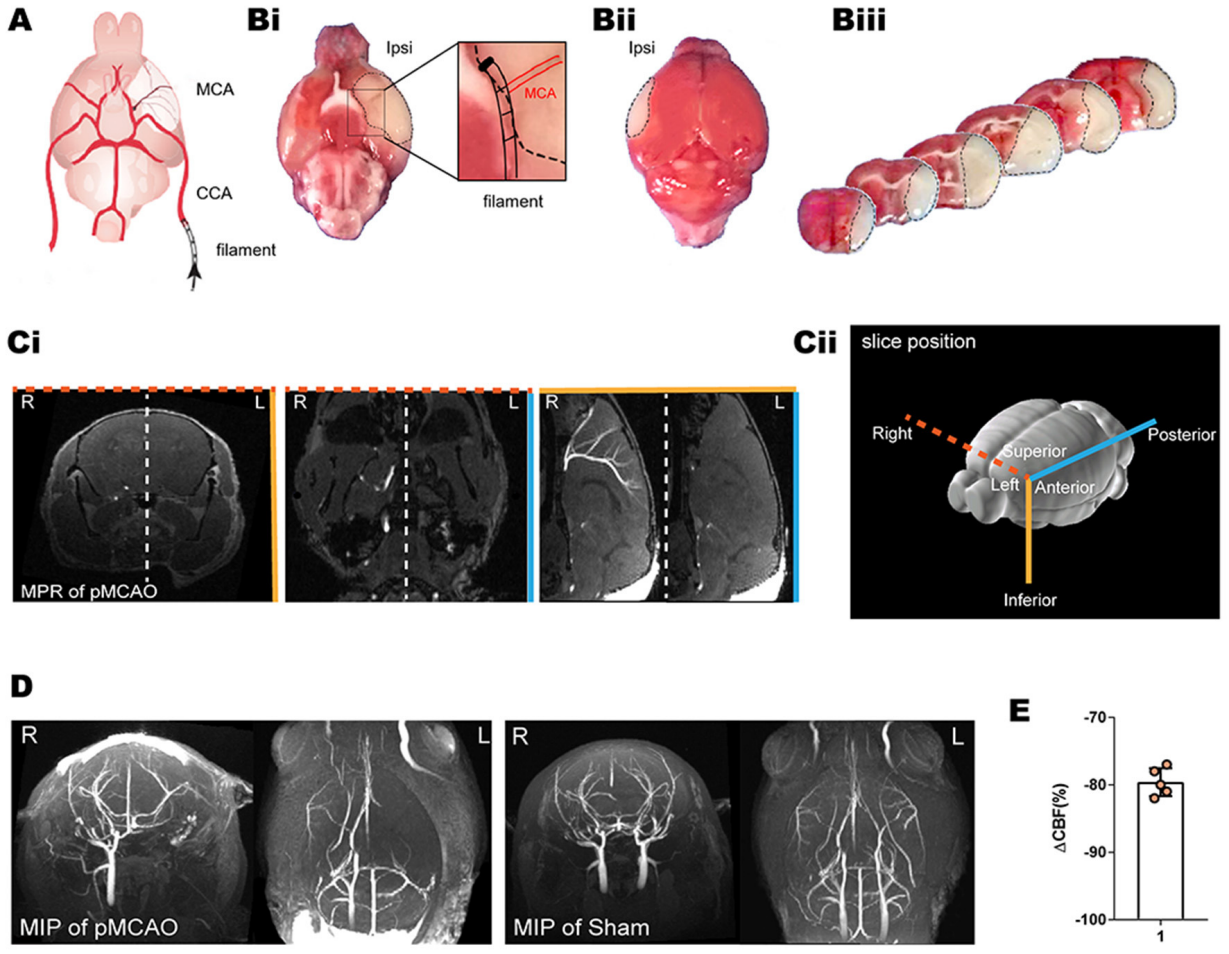
TTC staining, laser Doppler, and MRI of the pMCAO mice model. **(A)** Schematic diagram of the MCA occlusion. Reproduced with permission (10) **(Bi–iii)** Representative TTC staining of cerebral ischemia at 24 h after pMCAO. A lack of TTC staining was defined as infarction, whereas viable brain tissue was stained red. **(Ci,D)** The MCA occlusion displayed in coronal, axial, and sagittal planes of MPR and MIP. Bright signals are associated with the vasculature. L indicates the pMCAO side. **(Cii)** Schematic diagram of the slice positions of the MRI. Color-coded border lines denote the anatomical position of the slice. **(E)** Ipsilateral relative rCBF after MCAO ensured that the CBF decreased to below 25% of baseline as measured by laser Doppler.

To study the dynamics of glymphatic flow following cerebral edema, we induced pMCAO (Figure 1A) in the left (L) hemisphere of mice, examined ischemic regions using TTC staining 24 h after pMCAO (Figures 1Bi–iii). We confirmed the occlusion of MCA by multiplanar reconstructions (Figure 1C) and maximum intensity projections (Figure 1D) in 11.7 T MR images. In both images, pMCAO was represented by a unilateral loss of visible vasculature ipsilateral in the site of model induction. By contrast, bilateral vasculature could be observed in the sham-operated animals. The loss of vascular imaging markers was also quantified by calculating the CBF, as recorded by laser Doppler, to ensure that it was below 25% of baseline. pMCAO resulted in an immediate and strong reduction in the relative CBF (rCBF) (79.6% ± 2.1%; Figure 1E).

### *In vivo* Evaluation of Glymphatic Flow in Sham-Operated Mice

**Figure 2:**
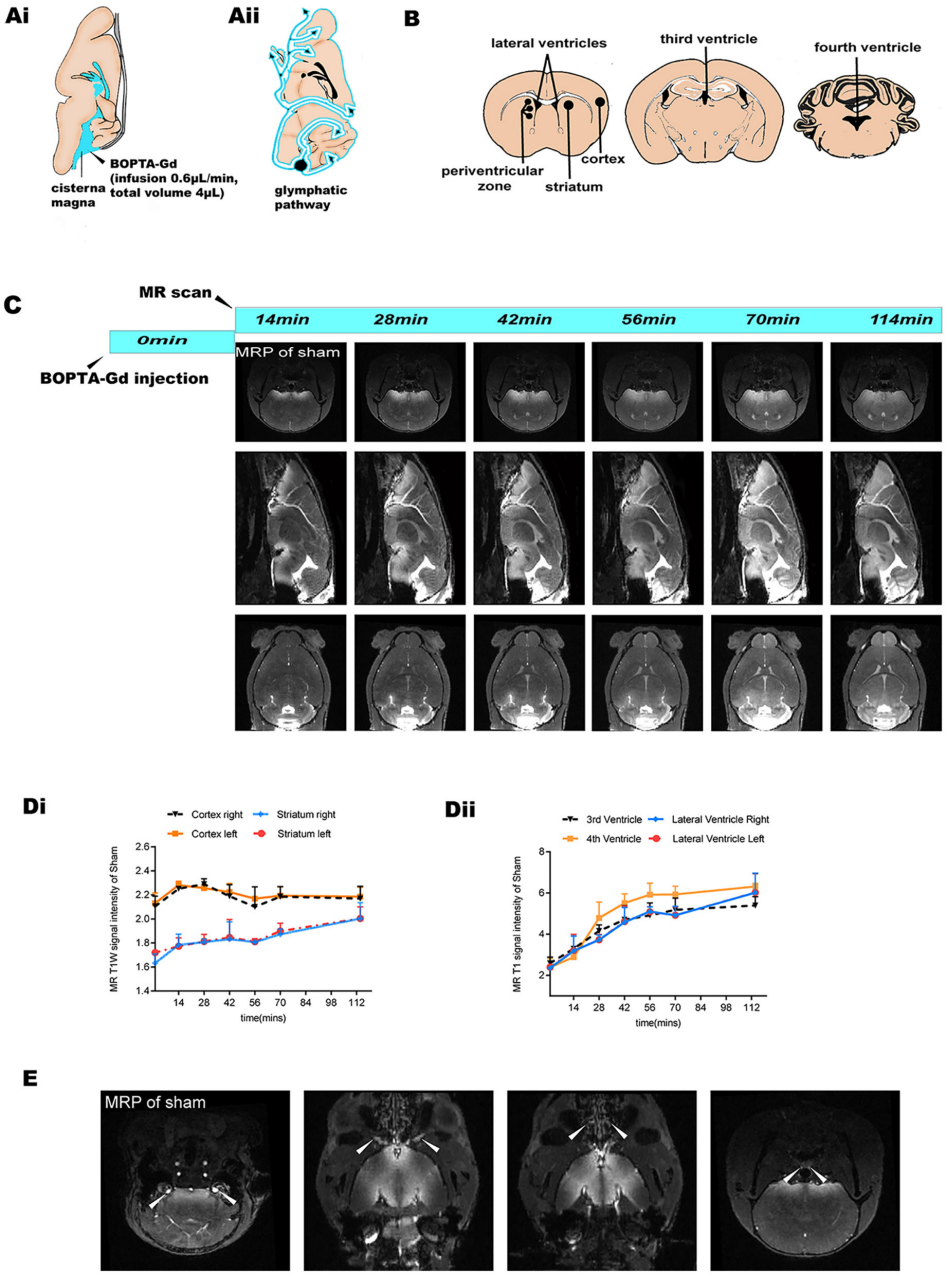
MRI-based evaluation of glymphatic perfusion. **(Ai,ii)** Schematic diagram of cisterna magna injection and the CSF drainage routes from the cisterna magna (alongside cerebral ventricles and ventral brain surface). **(B)** Schematic diagram of the approximate ROIs used for MRI quantification of SI, including the striatum, cortex, periventricular zone, and bilateral, third, and fourth cerebral ventricles. **(C)** Representative coronal, sagittal, and axial slices of CEMRI MRP images from the sham-operated group (BOPTA-Gd+), showing progressive enhancement of the brain parenchyma and cerebral ventricles. **(Di**,**ii)** Corresponding quantification of the SI from the sham-operation striatum, cortex, and cerebral ventricles. The average time evolution curves illustrate the dynamic of tissue uptake of BOPTA-Gd tracers (*n* = 3 per group). **(E)** Extracerebral CSF drainage routes revealed by the sham-operated group (BOPTA-Gd+). Arrows indicate cochleovestibular nerves, optic nerves, cribriform plate, and the peritracheal lymph nodes (from left to right, respectively).

We then investigated the glymphatic flow in sham-operated mice by *in vivo* CEMRI with BOPTA-Gd injection (BOPTA-Gd+) in the cisterna magna (Figure 2C). Sequential multiplanar reformation or reconstruction images revealed that gadolinium progressively entered the brain parenchyma, as shown in the schematic diagram in Figure 2A. The schematic diagram of the approximate ROIs used for MRI quantification is indicated in Figure 2B. In these images, an increase in SI, representing contrast agent uptake, can be observed as a function of time. Overall, the routes of CSF distribution were consistent with previous reports (Figure 2C) (26). Meanwhile, the contrast agent sequentially flowed from the cisterna magna to the bilateral, third, and fourth cerebral ventricles, as shown in Figure 2C. At 70 min postinjection, the brain ROI were completely perfused with the tracers (Figure 2C). The average time evolution curves illustrate the dynamics of tissue uptake of BOPTA-Gd tracers (Figure 2D). We also detected extracerebral CSF drainage routes at 14 min after BOPTA-Gd injection. Tracers drained from the subarachnoid space alongside the cranial nerves, such as the cochleovestibular and optic nerves (Figure 2E).

### Glymphatic Transport Kinetics Malfunction in the Acute Phase of pMCAO Mice

**Figure 3:**
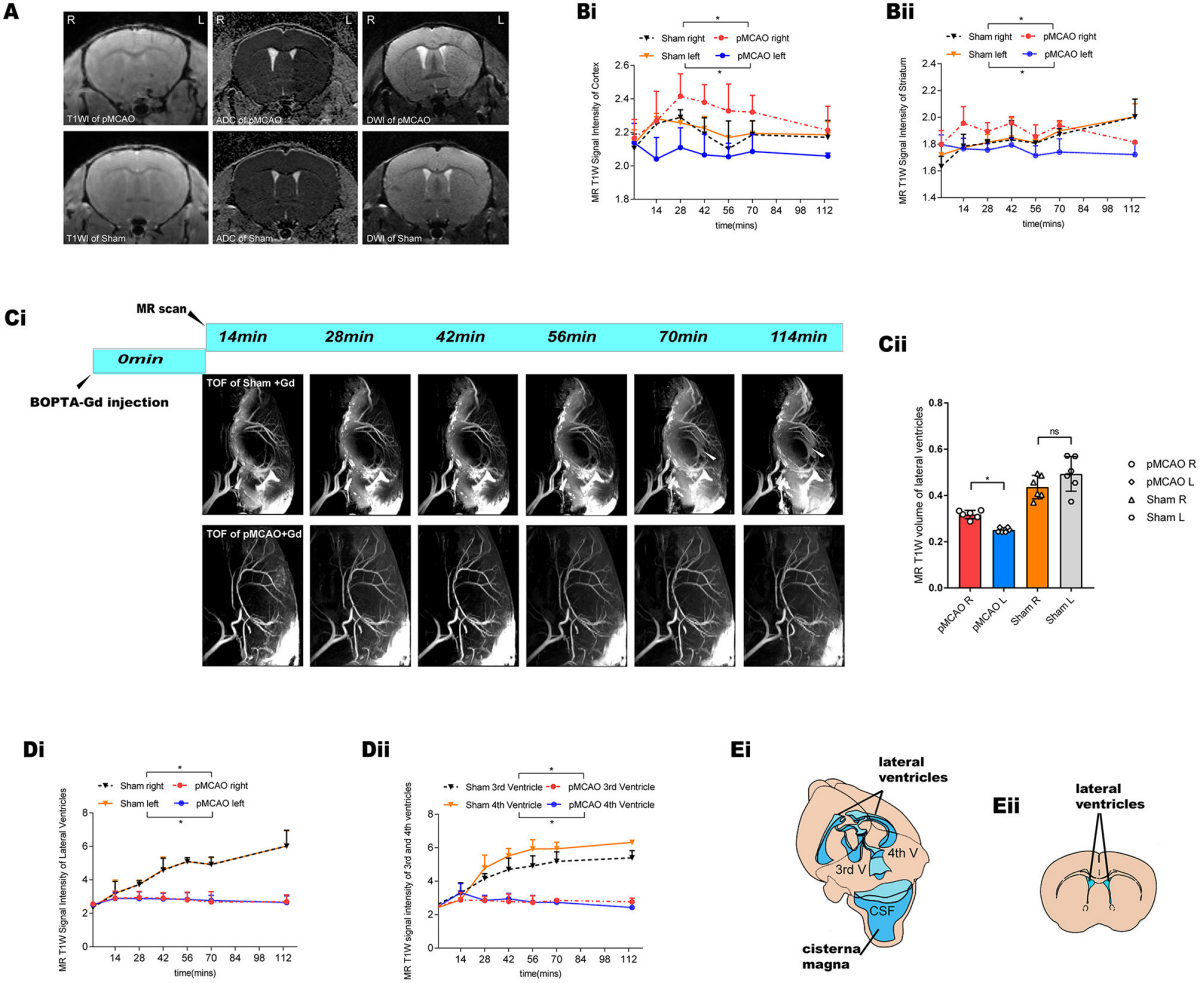
Glymphatic transport kinetics were impaired after the onset of ischemic edema in the pMCAO mice. **(A)** Representative T1W images, DWI, and ADC maps of pMCAO and sham-operated animals with BOPTA-Gd injection. The serial acquisition of MR images was performed at an interval of about 12 min and started at 28 min after pMCAO and 14 min after BOPTA-Gd injection. L indicates the pMCAO side. ROIs used for MRI quantification are as shown in **Figure 2B**. **(B,D)** The corresponding quantification of SI indicated the dynamic of tissue uptaking BOPTA-Gd tracers. Compared to the sham-operated animals, the T1W SI of the pMCAO group was decreased in the ipsilateral cortex/striatum **(Bi,ii)** and the bilateral cerebral ventricles **(Di,ii)** of the pMCAO brain (*n* = 3 per group). Concurrently, the increase in the SI of the contralateral cortex/striatum was detected by the enhancement of contrast agent, as compared to the sham-operated group. **(Ci)** Representative TOF images demonstrate that glymphatic flow was impaired in the bilateral ventricles of mice after the onset of ischemic edema in pMCAO with BOPTA-Gd injection. **(Cii)** Corresponding quantification of ventricular volume changes in the pMCAO and sham-operated animals, for which a decrease in the volume of the ipsilateral ventricles was observed. **(Ei)** Schematic diagram of the anatomical position of ventricles, namely, bilateral (LV), third (3V), and fourth (4V) cerebral ventricles. Reproduced with permission (10) **(Eii)** Blue-dashed areas indicate the approximate ROIs used for MRI quantification of the lateral ventricle volume.

Interestingly, in the MRI we noticed a blockage of the ipsilateral (L) parenchyma and the bilateral ventricle perfusion immediately after pMCAO (Figures 3A,Ci). By contrast, the inflow of contrast agent was observed in the contralateral (R) hemisphere in the pMCAO and the sham-operated animals, further suggesting that glymphatic flow was altered in this pMCAO mouse model. To quantify these effects, we calculated the corresponding SI in the cortex, striatum, and ventricles in the pMCAO and the sham-operated animals (Figures 3Bi,ii,Di,ii). We found that glymphatic perfusion of the ipsilateral cortex/striatum and bilateral cerebral ventricles of the pMCAO mice was reduced compared with sham-operated animals. By contrast, glymphatic perfusion in the contralateral cortex/striatum was increased. These results provide evidence of glymphatic perfusion from the cisterna magna to the cerebral ventricles. However, the volume of the ventricles (Figure 3Cii) was reduced in the ipsilateral pMCAO mice and was accompanied by a delayed influx in the bilateral ventricles (Figure 3D). The schematic diagram of the anatomical position of ventricles, and the approximate ROIs used for MRI quantification of the lateral ventricle volume are shown in Figure 3E.

### Analysis of the Link Between Ischemic Edema and Glymphatic Failure

**Figure 4:**
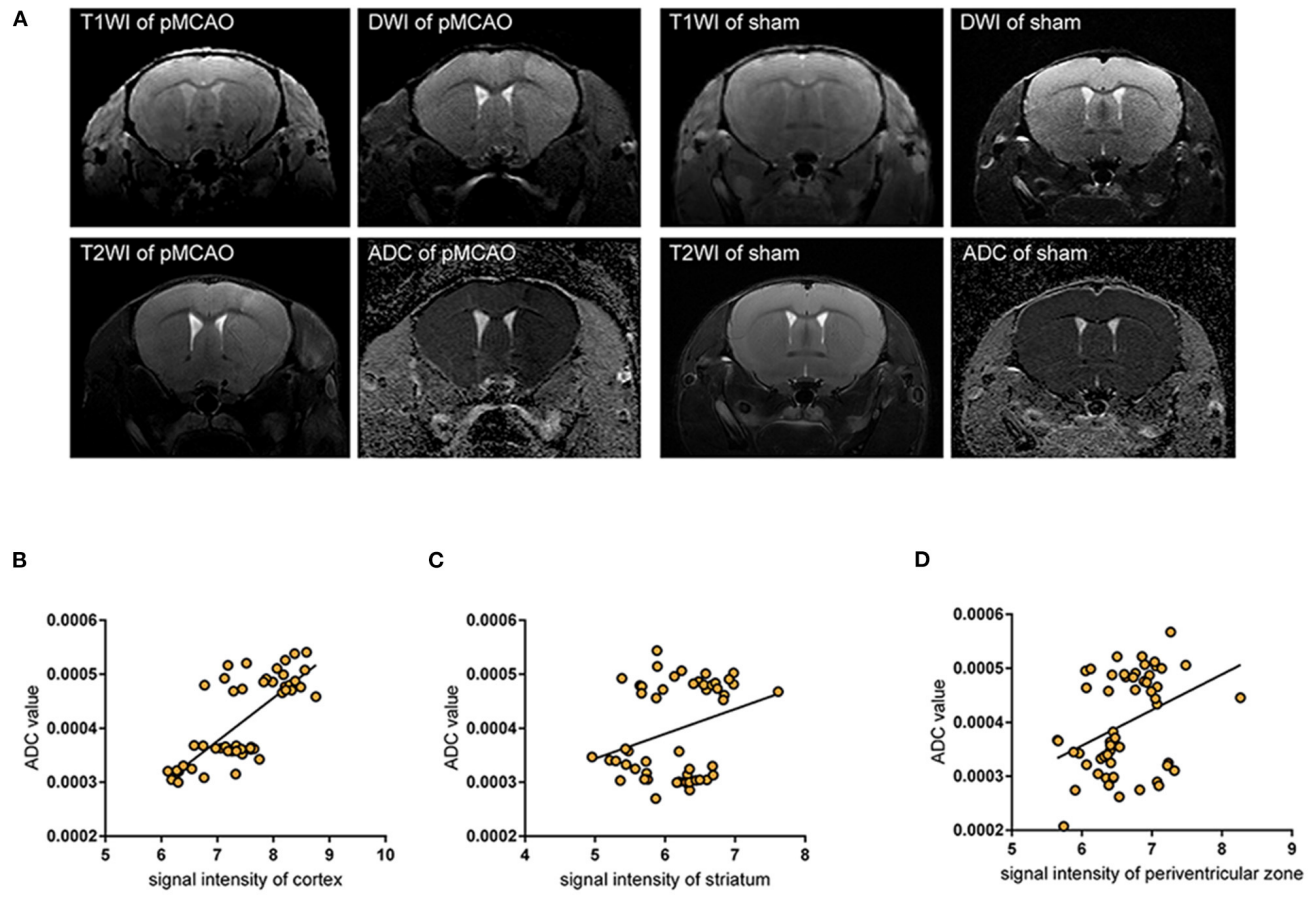
Impaired glymphatic kinetics were correlated to ischemic edema in pMCAO as evaluated by 11.7 T MRI. **(A)** Representative T1W images, DWI, and ADC maps of pMCAO and sham-operated animals with BOPTA-Gd injection, and T2W images of cerebral ischemia at ~150 + 28 min after pMCAO. L indicates the damaged side. **(B–D)** The corresponding quantifications of T1W SI in the cortex **(B)**, striatum **(C)**, and periventricular zone **(D)** were positively correlated to ADC values in pMCAO and the sham-operated animals by MR scan. The serial acquisition of MR images was performed by an interval of about 12 min, started at 28 min after pMCAO and 14 min after BOPTA-Gd injection. The correlation analysis indicated that the progression of ischemic edema was consistent with the impaired glymphatic transport kinetics after cytotoxic edema appeared, as evaluated by MRI.

To evaluate cerebral edema, we acquired T1WI, DWI, and ADC brain maps, these sequential scans were performed 28 min after pMCAO with 14 min BOPTA-Gd injections until 178 min (150 + 28 min) (*n* = 3 per group), serial acquisition of MR images was performed using an interval of about 12 min, and confirmed brain infarction by T2W images at ~178 min (Figure 4A). Accordingly, we acquired the T1W and ADC value brain maps sequentially in the sham-operation group 14 min after BOPTA-Gd injection. Glymphatic transport kinetics were determined based on the SI in T1WIs, while cytotoxic edema was identified as cases of DWI hyperintensity with reduced diffusion and with ADC dark signal (27). When we measured molecular motion with DWI, only the ADC value was calculated. We observed a positive correlation between SI in T1WIs and the corresponding ADC value in the cortex, striatum, and periventricular zone, suggesting that the impaired glymphatic transport kinetics in these regions are associated with the progression of pMCAO edema, as indicated by ADC values changes (Figures 4B–D).

### Immunofluorescent Analysis of AQP4 Expression and Polarization

**Figure 5:**
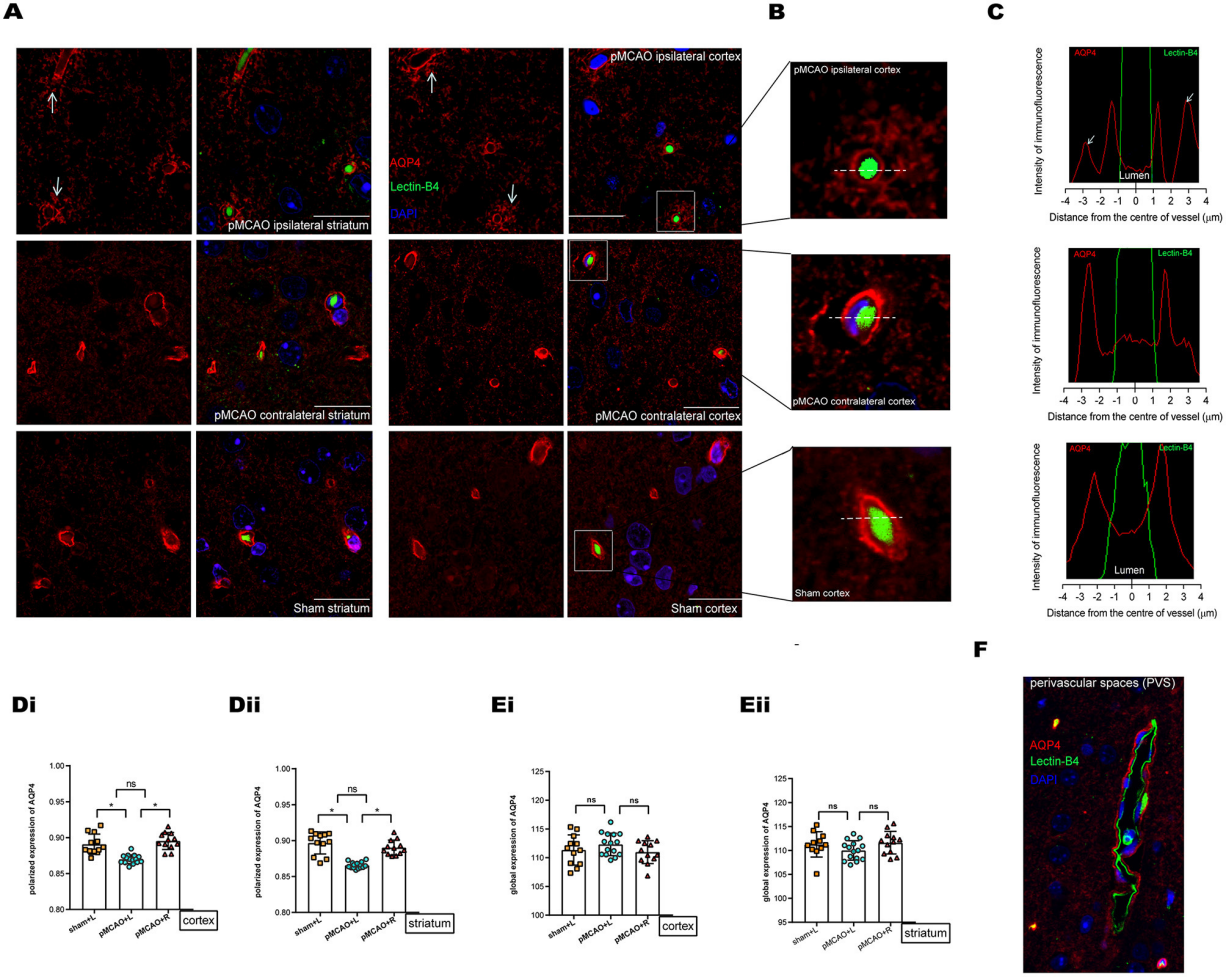
Immunofluorescent staining of AQP4 in the cortex and striatum of sham-operated and 150 min post-pMCAO animals. **(A)** Representative images of immunofluorescent staining of AQP4 in the cortex and striatum in 150 min post-pMCAO and sham-operated animals. The scale bar is 20 μm. Magnification is 100 ×. **(B)** Three representative magnified images in the boxed areas of **(A)** are shown as a representative expression pattern. **(C)** The axis perpendicular to blood vessels was used to quantify the expression across vessel cross-sections. AQP4 expression surrounding blood vessels in the ipsilateral cortex of pMCAO mice was decreased (depolarization expression pattern). **(D)** Quantification of at least 12 images (a minimum of 3 images per animal, *n* = 4) shows changes in AQP4 polarization. Compared with the sham-operated and contralateral pMCAO, polarized expression of AQP4 immunofluorescence was decreased surrounding blood vessels in the cortex and striatum of the ipsilateral pMCAO brain. **(E)** Quantification of at least 12 images (a minimum of 3 images per animal, *n* = 4) shows changes in global AQP4 expression. Compared with the sham-operated and contralateral pMCAO, global expression of AQP4 immunofluorescence was not significantly altered in the cortex and striatum of the ipsilateral pMCAO brains. **(F)** Representative image of immunofluorescent staining of PVS (red indicates AQP4, green indicates basement membrane, and blue indicates DAPI) in the sham-operated group.

Representative images of pMCAO and sham-operated animals (Figures 5A–C) showed an ischemia-affected ipsilateral (L) cortex and striatum 150 min after surgery. The vascular endothelial surface was delineated by I-B4 staining. At 150 min after pMCAO induction, parenchymal vessels in pMCAO animals exhibited endothelial IB4-FITC signals that did not differ from those of sham-operated animals (Figure 5B). This lack of difference indicated that the BBB remained intact (18, 21). In the current work, AQP4 was conjugated with Alexa Fluor 594, and nuclei were visualized with DAPI. AQP4 polarization levels were obtained by comparing the expression ratio of AQP4 at perivascular domains vs. parenchymal domains (25), which were analyzed on corresponding images obtained under 100 × magnification. Specifically, we compared the contralateral tissue 150 min after surgery in pMCAO and sham-operated animals. Overall, in pMCAO animals, we found no significantly altered global AQP4 expression (Figure 5E). Quantitative analysis showed that perivascular polarity of AQP4 decreased in ipsilateral pMCAO brains, with the most pronounced effects in the cortex and the striatum (Figure 5D). The widespread loss of perivascular AQP4 polarization accompanied the decline in glymphatic dynamics. Representative image of immunofluorescent staining of PVS in the sham-operated group is shown in Figure 5F.

### Immunohistochemical Analysis of AQP4 Expression in the Ependymal Wall

**Figure 6:**
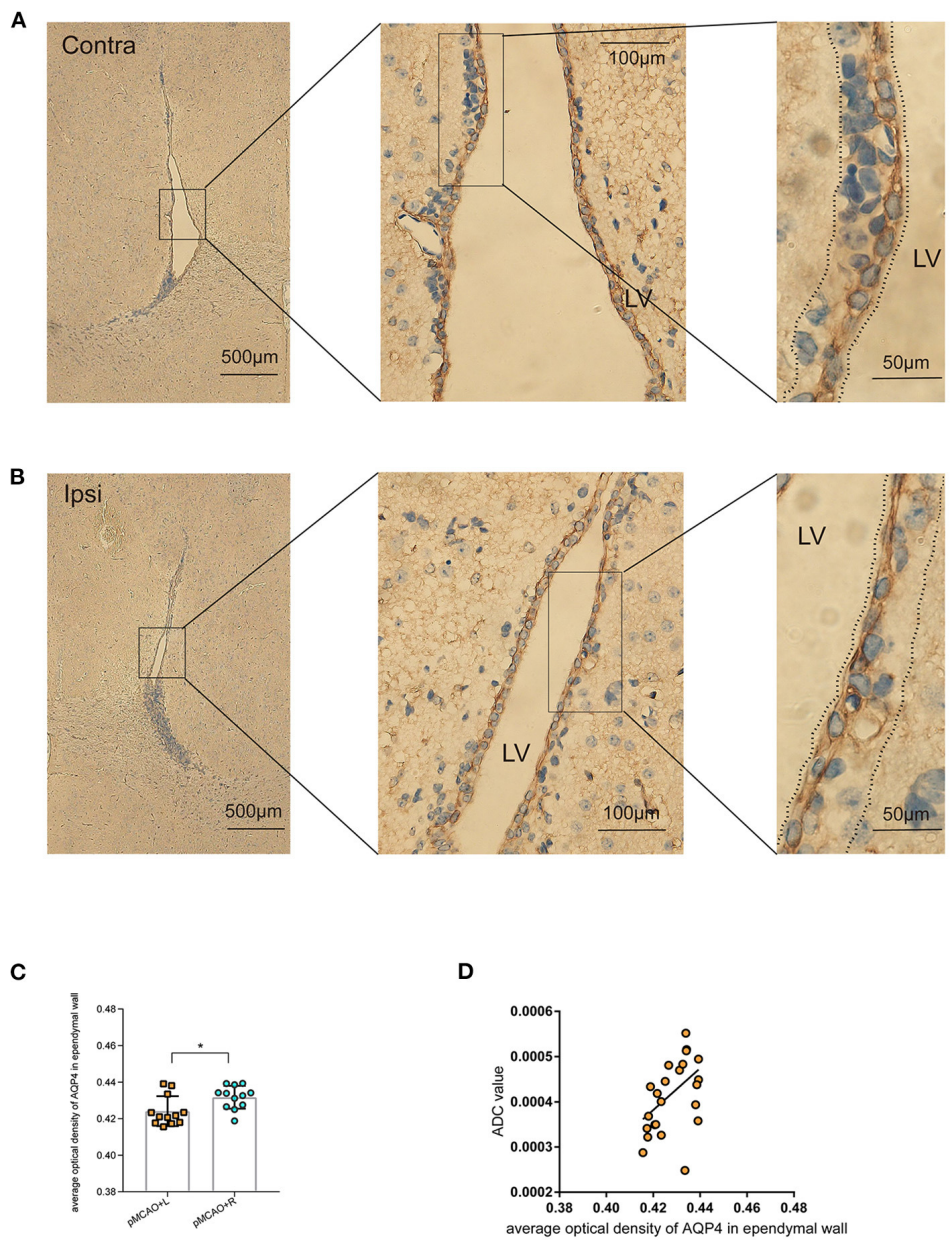
Immunohistochemical staining of AQP4 in the LV ependymal wall of 150 min post-pMCAO animals. **(A,B)** Representative images showing AQP4 immunohistochemical staining (brown) of coronal brain sections in the LV ependymal barrier of the bilateral brain in 150 min post-pMCAO animals. These images revealed comparable morphology of the bilateral ependyma walls outlining LV. Boxed areas are enlarged successively from left to right. The areas between the dotted lines were used to quantify AQP4 expression in the LV ependymal wall. The scale bars are indicated in each box, and the magnification is 4 ×, 40 ×, and 100 ×, respectively. **(C)** Quantification of at least 12 images (a minimum of three images per animal, *n* = 4) revealed changes in AQP4 expression. Compared with the contralateral pMCAO animals, AOD of AQP4 immunohistochemical staining was reduced in the LV ependymal wall of the ipsilateral pMCAO brain. **(D)** The corresponding ADC values in the periventricular zone of pMCAO animals at the 150 min MR scan were positively correlated to the AOD of AQP4 in the immunohistochemical staining of bilateral LV ependymal walls. ROIs used for MRI quantification are as shown in Figure 2B.

Representative images showing AQP4 immunohistochemical staining (brown) of coronal brain sections in the lateral ventricle (LV) ependymal barrier (Figures 6A,B) revealed that AQP4 expression was decreased in the LV ependymal wall of the ipsilateral pMCAO brain (Figure 6C). These images also revealed AOD values with no apparent abnormalities in ependymal architecture. Furthermore, we observed a positive correlation between the corresponding ADC values in the periventricular zone of pMCAO animals at the MR scan acquired after 150 min and the AOD of AQP4 in the immunohistochemical staining of bilateral LV ependymal walls (Figure 6D). This result suggests that decreased expression of AQP4 in ependymal walls was correlated to the diminishing of ADC values in the periventricular zone.

## Discussion

Our results demonstrate in a pMCAO model with an intact BBB (18, 21) that the accumulation of edema is not accompanied by the transendothelial extravasation water (28) across the BBB. Of note, at as early as 150 min post-pMCAO, in the period during which the transformation of cytotoxic edema to vasogenic edema occurs, the ischemic edema regions showed a depolarization of AQP4 in the PVSs. The key finding was that there was a positive correlation between T1WI SI and the corresponding ADC values in the cortex, striatum, and periventricular zone. This suggested that glymphatic transport kinetics were impaired after the ischemic edema appeared in pMCAO, were accompanied by depolarization of AQP4, and were correlated to the progression of edema.

Cerebrospinal fluid (CSF) can provide a predominant source for the initial rise in the brain water content of ischemic tissues. The glymphatic influx of CSF into the PVSs (9) of ischemic brain tissues is the critical cause of acute ischemic brain edema within ~5 min (10). Given the possible treatment strategies require the time for the operation to be executed, it is necessary to investigate the specific contribution of the glymphatic dynamics in different stages of acute ischemia edema. As such, we investigated the pattern of glymphatic flow that could account for the liquid drainage in the parenchyma and periventricular zone following induced acute ischemic brain edema as a follow-up study. Uptake of tracers and extracerebral CSF drainage routes have been observed in sham-operated animals as shown in Figures 2C,E. In the pMCAO model, we found that CSF dynamic flow was reduced in the ipsilateral brain parenchyma and accompanied by an increase in flow in the contralateral as indicated in Figure 3. These observations suggest that the accumulation of liquid in pMCAO brains may result from an impaired glymphatic system or be responsible for the suppression of the glymphatic dynamics. Glymphatic transport is driven by arterial pulsation (29, 30), and ISF drainage may be increased by vasomotion as a driving force, as observed by two-photon microscopy (31). Therefore, the regional differences in glymphatic flow observed here may be due to discrepancies in a convective flow drive mechanism. However, another study recently provided evidence that the directional movement of CSF in the PVS is not generated by arterial pulsations and can be explained by naturally occurring flow (32). Furthermore, contrast agents were found to rapidly enter the ventricular system in the sham-operated group (Figure 3), suggesting that cistern-labeled CSF could enter the ventricles. Previous data suggest a critical role of the ventricle in brain water drainage (33, 34). PVS pathways were found to connect different parts of the brain to the ventricles. In particular, a connection between the lateral ventricle and ventral brain surface has been identified through PVS pathways surrounding the anterior choroidal artery (33). Thus, there might exist a pathway that involves the transependymal exchange of CSF, followed by the clearance therefore from the parenchyma *via* a mechanism similar to the glymphatic system.

As a key determinant of the glymphatic system, AQP4 is a transmembrane and bidirectional water channel that is highly expressed on astrocytes, particularly at the sites of fluid transport at the pial surrounding cerebral blood vessels and ependymal surfaces in contact with the CSF in the ventricular system, and acts as a facilitator for water transport both in cells and tissues (16, 35–37). In a recent study, CSF was identified as a major source of water that drives directly AQP4-dependent edema, which acts as a consequence of CSF and brain ISF exchange (10). Other studies suggested a controversial opinion, in that the depletion of astrocytic AQP4 caused water to accumulate in the brain (34) and markedly impaired interstitial solute clearance (38). Moreover, recent studies on AQP4-knockout mice have suggested that the water movement in the paravascular space is mediated through diffusion and convective flow (39, 40), and elucidated the concept that AQP4 plays a vital role in the central nervous system (CNS) water homeostasis. In this sense, AQP4 is thought to facilitate the development or clearance of CNS edema. Previous studies have shown the glymphatic clearance function depends on the highly selective expression of AQP4 in microvascular end-feet of astrocytes in the aging brain, traumatic brain injury, and animal models of cerebral small vessel disease (38, 41, 42). We explored here the loss of polarized AQP4 expression leads to compromised glymphatic activity as soon as edema appeared at 150 min post-pMCAO (Figure 5). We also observed a decrease in AQP4 expression in the LV ependymal wall of the ipsilateral pMCAO brain, accompanied by a reduced volume of the ventricles and a delay in CSF uptake from the cisterna magna, as shown in Figures 3C, 6C. The participation of AQP4 in structural function, such as the distensibility capacity of the ventricular system, is also related to CSF homeostasis by altering the CSF drainage and the ventricular compliance in AQP4 mutation mice (43). Aquaporins (AQPs) line the periventricular wall. Previous studies have hypothesized that AQP4 participates in the development and integrity of the ependyma, however, the underlying mechanism of this effect has not been identified (44).

Cerebral edema is primarily an intracellular water accumulation process, caused by water influx through AQP4 passively in response to osmotic gradients (16), and usually as a consequence of ischemia-hypoxia. Of all the cell types susceptible to cytotoxic edema in the cerebral nervous system, astrocytes are particularly vulnerable (28). Along these lines, a recently proposed treatment targeting the mechanism of CNS edema is supported by the idea that AQP4 localization is also dynamically regulated at the subcellular level, from intracellular vesicles to the plasma membrane, affecting membrane water permeability (45). Due to the described dynamic subcellular changes of aquaporin abundance (45, 46), and there being highly polarized at the fluid interface (15), the membrane permeability and either the influx of CSF or ISF dynamics must be selective or suppressed. AQPs have been validated as an important drug target but there is no single drug that has yet been approved to successfully target it, it is hard to target edema and the glymphatic function using one line of therapies (47, 48). The previous studies have shown an increased AQP4 membrane localization in primary human astrocytes which was not accompanied by a change in AQP4 protein expression levels (45, 46). This subcellular mislocalization can be a potential therapeutic target. More studies to investigate the communication between AQPs (mainly AQP4) and other therapeutic targets are required to identify AQP modulators. Targeting the ion channel function and regulation mechanisms of AQP4 subcellular relocalization provides new insights into drug development (49). Currently, chelation of Ca^2+^ or CaM inhibition through egtazic acid acetoxymethyl ester (EGTA-AM) (45) or trifluoperazine (TFP) (50) had been reported to effectively reduce ischemia CNS edema.

In conclusion, glymphatic transport kinetics were suppressed between the onset of cytotoxic edema and the disruption of the BBB. These impaired glymphatic transport kinetics were correlated to the diminishing of ADC values that vary depending on edema progression, as measured by MRI, and that are associated with the depolarization of AQP4 in both the parenchyma PVSs and periventricular zone. As such, targeting the dynamic subcellular relocalization of the water channel protein AQP4, and improving glymphatic dynamics by promoting polarization of AQP4 may alleviate acute ischemic cerebral edema and similar pathologies.

## Data Availability Statement

The raw data supporting the conclusions of this article will be made available by the authors, without undue reservation.

## Ethics Statement

The animal study was reviewed and approved by Huashan Hospital Institutional Review Board.

## Author Contributions

JZ, WC, HW, HZ, YX, and GF performed the experiments, participated in data collection, and revision of this manuscript. WC, HW, and QD designed the study. HZ and YX analyzed the data. JZ drafted this manuscript and prepared figures. All authors contributed to the article and approved the submitted version.

## Funding

This study was supported by the National Nature Science Foundation of China (Nos. 81870915 and 82071197).

## Conflict of Interest

The authors declare that the research was conducted in the absence of any commercial or financial relationships that could be construed as a potential conflict of interest.

## Publisher's Note

All claims expressed in this article are solely those of the authors and do not necessarily represent those of their affiliated organizations, or those of the publisher, the editors and the reviewers. Any product that may be evaluated in this article, or claim that may be made by its manufacturer, is not guaranteed or endorsed by the publisher.
