# Corrigendum: Impaired Glymphatic Transport Kinetics Following Induced Acute Ischemic Brain Edema in a Mouse pMCAO Model

**DOI:** 10.3389/fneur.2022.929798

**Published:** 2022-05-16

**Authors:** Jianying Zhang, Hongchen Zhao, Yang Xue, Yiqi Liu, Guohang Fan, He Wang, Qiang Dong, Wenjie Cao

**Affiliations:** ^1^Department of Neurology, Huashan Hospital, Fudan University, Shanghai, China; ^2^The Key Laboratory of Computational Neuroscience and Brain-Inspired Intelligence, Institute of Science and Technology for Brain-Inspired Intelligence, Fudan University, Shanghai, China; ^3^State Key Laboratory of Medical Neurobiology, Fudan University, Shanghai, China; ^4^National Clinical Research Center for Aging and Medicine, Huashan Hospital, Fudan University, Shanghai, China

**Keywords:** magnetic resonance imaging, glymphatic, aquaporin-4, cerebral ischemic edema, polarization

In the published article, information was omitted from the captions for **Figure 1A** and **Figure 3Ei** as published. The relevant content permissions and attributions were not indicated in the figure captions, only listed in the references. The corrected captions appear below.

**Figure 1**. TTC staining, laser Doppler, and MRI of the pMCAO mice model. **(A)** Schematic diagram of the MCA occlusion. Reproduced with permission (10) **(Bi–iii)** Representative TTC staining of cerebral ischemia at 24 h after pMCAO. A lack of TTC staining was defined as infarction, whereas viable brain tissue was stained red. **(Ci,D)** The MCA occlusion displayed in coronal, axial, and sagittal planes of MPR and MIP. Bright signals are associated with the vasculature. L indicates the pMCAO side. **(Cii)** Schematic diagram of the slice positions of the MRI. Color-coded border lines denote the anatomical position of the slice. **(E)** Ipsilateral relative rCBF after MCAO ensured that the CBF decreased to below 25% of baseline as measured by laser Doppler.

**Figure 3**. Glymphatic transport kinetics were impaired after the onset of ischemic edema in the pMCAO mice. **(A)** Representative T1W images, DWI, and ADC maps of pMCAO and sham-operated animals with BOPTA-Gd injection. The serial acquisition of MR images was performed at an interval of about 12 min and started at 28 min after pMCAO and 14 min after BOPTA-Gd injection. L indicates the pMCAO side. ROIs used for MRI quantification are as shown in **Figure 2B**. **(B,D)** The corresponding quantification of SI indicated the dynamic of tissue uptaking BOPTA-Gd tracers. Compared to the sham-operated animals, the T1W SI of the pMCAO group was decreased in the ipsilateral cortex/striatum **(Bi,ii)** and the bilateral cerebral ventricles **(Di,ii)** of the pMCAO brain (*n* = 3 per group). Concurrently, the increase in the SI of the contralateral cortex/striatum was detected by the enhancement of contrast agent, as compared to the sham-operated group. **(Ci)** Representative TOF images demonstrate that glymphatic flow was impaired in the bilateral ventricles of mice after the onset of ischemic edema in pMCAO with BOPTA-Gd injection. **(Cii)** Corresponding quantification of ventricular volume changes in the pMCAO and sham-operated animals, for which a decrease in the volume of the ipsilateral ventricles was observed. **(Ei)** Schematic diagram of the anatomical position of ventricles, namely, bilateral (LV), third (3V), and fourth (4V) cerebral ventricles. Reproduced with permission (10) **(Eii)** Blue-dashed areas indicate the approximate ROIs used for MRI quantification of the lateral ventricle volume.

The authors apologize for this error and state that this does not change the scientific conclusions of the article in any way. The original article has been updated.

## Publisher's Note

All claims expressed in this article are solely those of the authors and do not necessarily represent those of their affiliated organizations, or those of the publisher, the editors and the reviewers. Any product that may be evaluated in this article, or claim that may be made by its manufacturer, is not guaranteed or endorsed by the publisher.
